# Unrecognised cardiovascular disease in type 2 diabetes: is it time to act earlier?

**DOI:** 10.1186/s12933-018-0788-7

**Published:** 2018-11-21

**Authors:** Guntram Schernthaner, Chaim Lotan, Elina Baltadzhieva-Trendafilova, Jonas Ceponis, Martin Clodi, Kristine Ducena, Eva Goncalvesova, Cristian Guja, Marek Honka, Andrej Janež, Nebojša Lalić, Roger Lehmann, Noémi Nyolczas, Priit Pauklin, Andrzej Rynkiewicz, Igor Sergienko, Lea Smirčić Duvnjak

**Affiliations:** 1 Medical University of Vienna, Vienna, Austria; 2 Hadassah-Hebrew University Medical Center, Jerusalem, Israel; 3 National Cardiology Hospital, Sofia, Bulgaria; 4 Institute of Endocrinology, Medical Academy, Lithuanian University of Health Sciences, Kaunas, Lithuania; 5 Department of Internal Medicine, St. John Hospital, Linz, Austria; 6 Faculty of Internal Medicine, University of Latvia, Riga, Latvia; 7 Department of Heart Failure and Transplantation, National Institute of Cardiovascular Diseases, Bratislava, Slovak Republic; 8 Department of Diabetes, Nutrition and Metabolic Diseases, Carol Davila University of Medicine and Pharmacy, Bucharest, Romania; 9 Fakultní Nemocnice Ostrava, Poruba, Czech Republic; 10 National Institute of Endocrinology and Diabetology, Lubochna, Slovakia; 11 Clinic for Endocrinology, Diabetes and Metabolic Diseases, CCS, Faculty of Medicine, University of Belgrade, Belgrade, Serbia; 12 Division of Endocrinology and Diabetes of the University Hospital, Zurich, Switzerland; 13 Department of Cardiology, Hungarian Defence Forces-Medical Centre, Budapest, Hungary; 14 Department of Cardiology, Tartu University Hospital, Tartu, Estonia; 15 Department of Cardiology and Cardiosurgery, School of Medicine, University of Warmia and Mazury, Olsztyn, Poland; 16 Russian Cardiology Research Complex, Moscow, Russia; 17 Department of Endocrinology and Metabolic Diseases, Vuk Vrhovac University Clinic for Diabetes, Endocrinology and Metabolic Diseases, Merkur University Hospital, School of Medicine, Endocrinology and Metabolic Diseases, University of Zagreb, Zagreb, Croatia

**Keywords:** Type 2 diabetes, Cardiovascular disease, Silent, Asymptomatic, Unrecognised, Atypical, Screening

## Abstract

Cardiovascular disease (CVD) is the most significant prognostic factor in individuals with type 2 diabetes (T2D). However, a significant number of individuals may develop CVD that does not present with the classic angina-related or heart failure symptoms. In these cases, CVD may seem to be ‘silent’ or ‘asymptomatic’, but may be more accurately characterised as unrecognised diabetic cardiac impairment. An initial step to raise awareness of unrecognised CVD in individuals with T2D would be to reach a consensus regarding the terminology used to describe this phenomenon. By standardising the terminologies, and agreeing on the implementation of an efficient screening program, it is anticipated that patients will receive an earlier diagnosis and appropriate and timely treatment. Given the availability of anti-diabetic medications that have been shown to concomitantly reduce CV risk and mortality, it is imperative to improve early identification and initiate treatment as soon as possible in order to enable as many patients with T2D as possible to benefit.

## Introduction

Cardiovascular disease (CVD) has been shown to have a significantly high prevalence, incidence and mortality in individuals with type 2 diabetes (T2D). Moreover, patients with T2D may have seemingly asymptomatic CV damage [1, 2], with ischaemic episodes remaining undetected at a reported prevalence of one in three patients with diabetes (compared with one in five patients without diabetes) [3]. Therefore, CV sequelae may develop much earlier than detected, with the disease only recognised once symptoms are more pronounced [2, 4]. At a regional expert meeting convened to consider the challenges presented by unrecognised CVD in T2D, we identified a lack of clarity with regard to definitions, diagnostic criteria, prevalence and care. The atypical symptoms of CVD experienced by patients with T2D have variously been described as ‘silent’ or ‘asymptomatic’. Without a clear terminology, and with a paucity of recent studies, an assessment of prevalence is challenging, while guidelines have to rely on data obtained without uniform definitions and before the development of current standards of care. In light of this, we would like to propose a definition for unrecognised CVD in patients with T2D, as an initial step towards providing clarity on the disease, the atypical symptoms that healthcare professionals should be aware of, and how best to assess patients for unrecognised CVD. We also seek to offer a vision for how an improved definition could ultimately lead to a simple and readily implementable screening strategy.

## T2D as a risk factor for CVD

The association between CVD and T2D has long been established, with CVD remaining the principal cause of death and major source of disability among individuals with T2D, in part due to an exacerbation of mechanisms that underlie atherosclerosis and heart failure. Types of CVD with increased prevalence in patients with T2D include peripheral arterial disease, ischaemic stroke, stable angina, heart failure and non-fatal myocardial infarction [5]. A recent meta-analysis of 43 studies found that diabetes was adversely associated with long-term survival and risk of hospitalisation in 380,000 patients with acute and chronic heart failure (the prevalence of diabetes in the cohort was 26%) [6].

There is also evidence to suggest that for a proportion of patients with T2D the occurrence of CVD may go unrecognised [1, 2]. Often, patients with T2D report only weakness and shortness of breath during exertion, which might in many cases mask significant CVD. So-called ‘silent’ myocardial infarction occurs in approximately 20% of patients with T2D [4], and is associated with a poor prognosis [4], while evidence of ‘silent’ ischaemia is seen in the electrocardiograms of 34% of patients with T2D, compared with only 19% of controls (*p *< 0.02) [1]. Indeed, the atypical symptoms associated with unrecognised CVD can result in patients not receiving appropriate medical treatment that may be needed to prevent subsequent adverse outcomes, including those that occur from excessive physical effort.

**Fig. 1:**
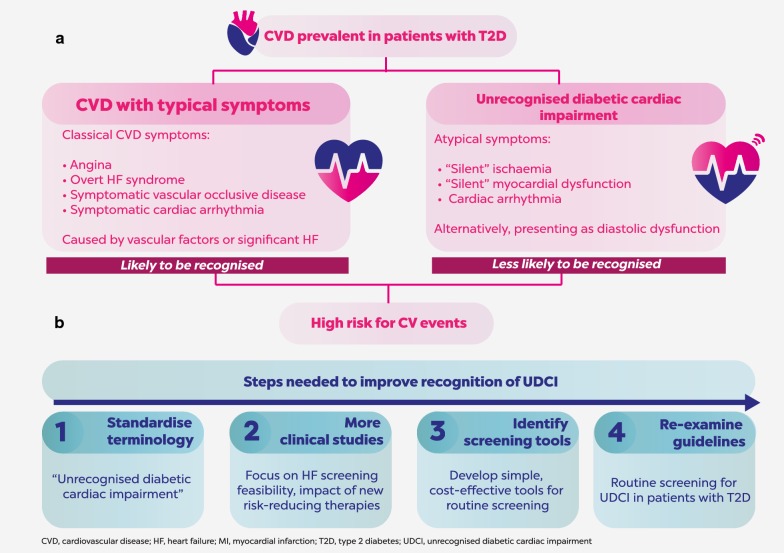
Defining and addressing UDCI. **a** Cardiovascular disease (CVD) is prevalent in patients with type 2 diabetes (T2D). In some cases, symptoms may be atypical (including patients with diastolic dysfunction) and the disease may therefore not be readily recognised, even though a high risk for CV events may nevertheless still be present. We propose the terminology “unrecognised diabetic cardiac impairment (UDCI)” for CVD with atypical symptoms in patients with T2D. **b** The introduction of a standardised terminology is the first of several steps that we believe will improve recognition of UDCI. New clinical studies, a re-examination of guidelines and the establishment of cost-effective, simple screening strategies will be important future steps

The mechanisms that underlie the atypical nature of CVD symptoms in some patients with T2D require further investigation. Whereas ischaemia-related symptoms may result from classic vascular risk factors associated with T2D, atypical symptoms (Fig. 1a) may instead be due to impaired pain sensation [2] or other yet undefined mechanisms. Diabetic cardiac autonomic neuropathy (CAN), which may affect up to 34% of patients with T2D, may cause abnormalities in heart rate control and vascular dynamics that can result in exercise intolerance, orthostatic hypotension, asymptomatic ischaemia and painless myocardial infarction [7]. CAN has been associated with increased prevalence of arrhythmia, sudden death and left ventricular diastolic dysfunction (LVDD) in patients with T2D; LVDD can progress to heart failure with preserved ejection fraction, with increased morbidity and mortality [7]. A recent observational study in patients with T2D, in addition to at least one additional CV risk factor and LDL cholesterol < 3.35 mmol/L, found that silent myocardial ischaemia was associated with risk factors that included atherogenic dyslipidaemia (triglycerides ≥ 2.26 mmol/L and HDL cholesterol ≤ 0.88 mmol/L), peripheral occlusive arterial disease, retinopathy and male sex [8].

## Current challenges in diagnosing and managing unrecognised CVD in T2D

At the present time, the phenomenon of unrecognised CVD in individuals with T2D is under-reported in the literature. There is a paucity of good quality, large-scale studies that fully explore the prevalence, incidence and prognostic potential of unrecognised CVD [2]. What studies do exist predate the possibility to provide DES as an invasive intervention [2] and current optimised care based on an understanding of cardioprotective effects with certain antidiabetic therapies.

The quality of evidence upon which current recommendations for screening asymptomatic patients are based is therefore limited, both in the power of the studies considered and in predating the availability of newer, innovative anti-diabetic drugs that have been demonstrated to improve CV outcomes in patients with T2D and CVD. This is particularly pertinent given that American Diabetes Association (ADA) recommendations on screening asymptomatic patients [9] were based on the findings of clinical studies that did not include these therapies. The European Society of Cardiology (ESC)/European Association for the Study of Diabetes (EASD) 2013 Guidelines on diabetes, prediabetes and CVD advised screening in selected patients, but identified a need to better characterise which patient will benefit [2, 10]. Thus, readily implementable guidance on how best to screen for unrecognised CVD is also needed.

A recent regional meeting of experts from 15 countries in Central and Eastern Europe convened to discuss the problem of unrecognised CVD in patients with T2D. There was unanimous agreement among the experts present that the changing treatment landscape provides an impetus for reconsidering current screening guidelines, and that the failure of the ADA guidelines to recommend screening of so-called “asymptomatic patients” with high atherosclerotic CV disease risk should be re-examined (Fig. 1b). When considering approaches to screening, the majority of experts who were present agreed that CV risk calculation systems, such as the American College of Cardiology and American Heart Association Atherosclerotic CVD Risk Calculator, should form part of patient evaluations.

The meeting also identified an initial challenge in agreeing on standardised definitions and terminology to describe unrecognised CVD: terms such as ‘silent’ and ‘asymptomatic’ have been used interchangeably when discussing this phenomenon—neither of which may be an accurate description, as CVD can arguably never be truly asymptomatic. Instead, “unrecognised diabetic cardiac impairment (UDCI)” is proposed as a standardised terminology that accurately accounts for the phenomenon of ‘asymptomatic’ CVD that is unrecognised in patients with T2D.

By standardising the way in which unrecognised CVD is defined, discussed and described, it is hoped that awareness of this most important prognostic factor of T2D will be increased among both patients and healthcare professionals. A more consistent use of terminology in research will enable improved evaluation of the prevalence and manifestation of UDCI in patients with T2D. Such increased awareness should drive the development of tools that may be used for effective, non-invasive patient screening, concurrent with a re-examination of guidelines in order to acknowledge the need for such screening. In time, we envisage that screening of patients will become a more accepted and routine part of regular care, with earlier identification of CVD enabling more patients to benefit from early treatment, including with anti-diabetic therapies that offer a concomitant reduction in CV morbidity and mortality.

Arriving at a standardised terminology is only the first step in a complex set of challenges. It is vital that there is increased awareness of UDCI amongst both patients with T2D and the healthcare professionals managing their treatment. Additional clinical studies are required to examine the true prevalence of UDCI, and to define the key risk factors and atypical symptoms that characterise this phenomenon. As research progresses, best practice recommendations will need to be developed for identifying and managing UDCI. Importantly, optimal screening and treatment strategies should take into account key criteria such as cost effectiveness for healthcare systems and the availability of anti-diabetic treatments with a proven ability to reduce CV morbidity and mortality.

## Defining unrecognised CVD in T2D

Given the variety of terms currently employed when discussing unrecognised CVD in T2D, it is unsurprising that this phenomenon is not widely recognised or appreciated. By establishing a consensus with regard to the terminology used, it should be possible to ensure that no pertinent data relating to this condition are overlooked owing to the use of an unfamiliar name. We would propose that ‘silent’ or ‘asymptomatic’ CVD in individuals with T2D should be referred to as ‘unrecognised diabetic cardiac impairment’ (UDCI), thereby highlighting the hidden nature of this condition, together with the link between atypical symptoms and diabetes (Fig. 1).

We acknowledge the importance of all types of unrecognised CV risk within the T2D patient population, including ischaemia and diastolic dysfunction (DD), which can lead to heart failure. Therefore, we propose that initial efforts to develop our understanding of UDCI should include, in addition to ischaemia, assessment of left ventricular dysfunction and/or heart failure (Fig. 1b). We expect that this pragmatic approach is most likely to yield success, for several reasons. First, there is a wide acceptance that heart failure is a major comorbidity associated with T2D. Second, there are a range of simple and cost-effective screening strategies and tools to investigate the risk of unrecognised heart failure as a component of UDCI among individuals with T2D. Third, SGLT2 inhibitors, a new class of anti-hyperglycaemic therapies, have shown promise in reducing the risk of hospitalisation for heart failure among patients with T2D, which has prompted ongoing clinical studies with pre-specified HF outcomes [10]. This emerging evidence was recognised by the 2016 ESC Guidelines for the diagnosis and treatment of acute and chronic heart failure (specifically, for empagliflozin based on the results of the EMPA-REG OUTCOME^®^ trial) [10], and suggests the potential for a positive effect on healthcare spending with appropriate early detection and treatment of conditions with a high risk for developing heart failure.

A relevant consideration for heart failure is that its prevalence is particularly high in elderly patients, and that the number of elderly patients with diabetes is increasing. We note that subgroup analyses of clinical studies have suggested that the potential of SGLT2 inhibitors to improve HF outcomes is retained even when looking only at this older population.

## Screening for UDCI

The existence of a clearly defined, widely accepted, standard terminology for UDCI should provide consensus within the medical community regarding when and how healthcare professionals should screen patients with T2D for CV risk. Standardisation of terminologies would provide clarity around this point and allow for the implementation of simple, readily available screening options that fulfil the following key criteria by being: cost effective; widely available; simple to administer and understand; capable of providing immediate patient feedback in order to facilitate more rapid decision making; and scope to permit the stratification of patients (Fig. 1b). A variety of tools are available that meet these criteria; these include the use of questionnaires to obtain detailed patient histories, digital data gathering using wearable personal health trackers, the stress test or 6-min walking test and the use of biomarkers such as B-type natriuretic peptide (BNP) or N-terminal pro BNP (Table 1). An echocardiogram may also be appropriate in suspected cases.

**Table 1:** Advantages and disadvantages of tools that might be used to screen patients with T2D for UDCI

Tool	Advantages	Disadvantages
Questionnaires	Can be carried out by a variety of people with limited affect to its validity and reliability Results can be quickly and easily quantified Can be analysed more ‘scientifically’ and objectively than other forms of research Can be used to compare and contrast data and to measure change	Patients may provide the answers that they think are expected Lacks validity It is difficult to assess how much thought a patient has given to each question The interpretation of each question may differ
Digital data gathering (e.g. wearable personal health tracker)	Provide continuous, objective, remote monitoring Patients can monitor and self-manage behaviours A significant volume of data can be captured Data collected may promote beneficial lifestyle changes	Accuracy may be affected by factors such as individual gait characteristics, body morphology, and where and how a device is worn on the body Patients may place greater faith in the accuracy of the device than is warranted Sensitive data may be captured with resultant privacy issues
Stress test or 6-minute walking test	Practical and simple requiring no specialised equipment Evaluates the global and integrated responses of all the systems involved during exercise Useful for measuring the response to medical interventions Findings are reproducible	Additional cardiopulmonary exercise testing may be required Adherence to strict protocols is required to ensure validity of data collected
Biomarkers	Free from recall bias Can provide sensitive and specific early detection of disease	Usually require a sample of body fluid to be taken Inter-individual variability may be a concern Specialised laboratory analysis may be required Reproducibility could be a concern May not be cost-effective

## Conclusions

Unrecognised CVD presents a significant burden in patients with T2D, and it is imperative that we improve the early identification and treatment of this vulnerable patient population. At the present time, a lack of clarity in terms of how unrecognised CVD is defined, diagnosed and treated, together with a paucity of relevant guideline recommendations, mean that patients may often be under-treated. We propose the introduction of UDCI as a standardised terminology, and call for a concerted effort to increase awareness of this condition and its consequences. By so doing, it should be possible to define the correct screening programme to facilitate early and appropriate treatment of patients with T2D and to minimise adverse CV outcomes.

## Abbreviations

ADA: American Diabetes Association
BNP: B-type natriuretic peptide
CAN: cardiac autonomic neuropathy
CVD: cardiovascular disease
ESC: European Society of Cardiology
T2D: type 2 diabetes
UDCI: unrecognised diabetic cardiac impairment
